# Prebiotic Synthesis of Aspartate Using Life’s Metabolism as a Guide

**DOI:** 10.3390/life13051177

**Published:** 2023-05-12

**Authors:** Stuart A. Harrison, William L. Webb, Hanadi Rammu, Nick Lane

**Affiliations:** Centre for Life’s Origins and Evolution (CLOE), Department of Genetics, Evolution and Environment, University College London, London WC1E 6BT, UK; w.webb.21@ucl.ac.uk (W.L.W.); hanadi.rammu.14@ucl.ac.uk (H.R.)

**Keywords:** protometabolism, origins of life, aspartate, oxaloacetate, pyridoxal, metabolism

## Abstract

A protometabolic approach to the origins of life assumes that the conserved biochemistry of metabolism has direct continuity with prebiotic chemistry. One of the most important amino acids in modern biology is aspartic acid, serving as a nodal metabolite for the synthesis of many other essential biomolecules. Aspartate’s prebiotic synthesis is complicated by the instability of its precursor, oxaloacetate. In this paper, we show that the use of the biologically relevant cofactor pyridoxamine, supported by metal ion catalysis, is sufficiently fast to offset oxaloacetate’s degradation. Cu^2+^-catalysed transamination of oxaloacetate by pyridoxamine achieves around a 5% yield within 1 h, and can operate across a broad range of pH, temperature, and pressure. In addition, the synthesis of the downstream product β-alanine may also take place in the same reaction system at very low yields, directly mimicking an archaeal synthesis route. Amino group transfer supported by pyridoxal is shown to take place from aspartate to alanine, but the reverse reaction (alanine to aspartate) shows a poor yield. Overall, our results show that the nodal metabolite aspartate and related amino acids can indeed be synthesised via protometabolic pathways that foreshadow modern metabolism in the presence of the simple cofactor pyridoxamine and metal ions.

## 1. Introduction

### 1.1. Life as a Guide to Protometabolism

All life shares common features implying a singular common ancestor of life on Earth. Typical features referenced in the literature include the genetic code [1,2,3], the structure of the ribosome [4,5] and the mechanism of transcription and translation [6,7]. The deep conservation of metabolic processes and reaction mechanisms, however, is often overlooked. While the wider structure of metabolism can vary considerably between the domains of life, a surprising number of invariant autotrophic pathways are conserved [8,9,10]. The chemical mechanisms that underpin metabolic pathways are often more conserved than the enzymes or the cofactors that facilitate them are [11].

Taken together, these observations suggest that the chemistry of metabolism may be more ancient than the enzymes that enact it, providing the simplest possible explanation for how biomolecules may have emerged at the origins of life on Earth. We refer to this hypothesis as the “protometabolism hypothesis” [12,13,14]. This hypothesis proposes that the chemical reactions underlying modern metabolism had direct continuity with geochemical and chemical processes on the early Earth, and that no reinvention of metabolism took place [15]. Rather, the later emergence of cofactors, genes and enzymes amplified an existing chemical network [16].

**Figure 1:**
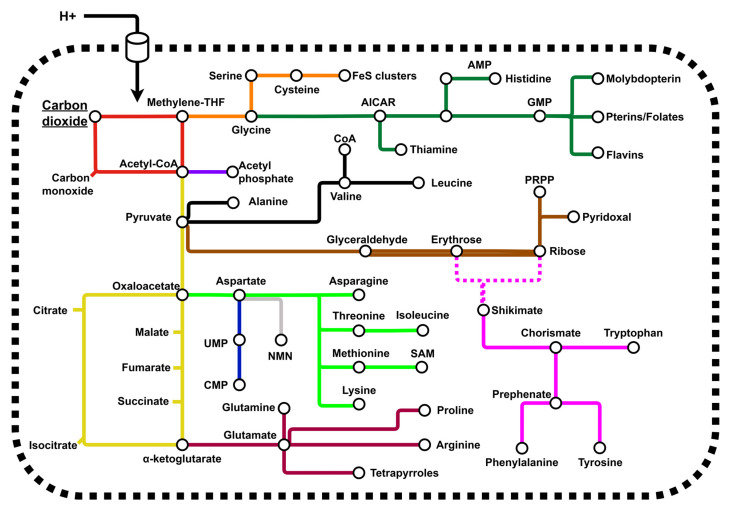
Minimal autotrophic protometabolism. The network presents a chemically focused set of reactions which have universal or ancient conservation across all domains of life. The network is oriented around a pH gradient-catalysed CO_2_ fixation pathway with chemical similarity to the acetyl-CoA pathway and extends to the essential amino acids, nucleotides, and cofactors required in modern metabolisms. Major super-pathways have been colour-coded, and dashed lines represent pathways with poor conservation between bacterial and archaeal clades.

A protometabolic hypothesis for the origins of life has gained traction in recent years. Prebiotic reactions analogous to core, autotrophic metabolic pathways have now been shown to take place under aqueous conditions, albeit with low efficiency [17,18,19,20,21,22,23,24,25,26,27,28,29]. Examples of such pathways include the acetyl-coA pathway, reactions of the Krebs cycle and parts of the pentose phosphate pathway. This is consistent with the idea that natural selection later amplifies flux through the protometabolic network, increasing yields [30,31]. These reactions are often found to occur with simple metal-ion catalysts, species agreed to be essential and abundant in many origins of life scenarios [32,33,34,35]. This hypothesis is currently in its early stages, with disparate syntheses of each species, missing reaction steps and minimal evidence for a continuous network that can be supported in a far-from-equilibrium system. The end goal is to identify a chemical system capable of mimicking conserved pathways of metabolism and that can synthesise the universal and essential biomolecules, notably cofactors, nucleotides, and amino acids (Figure 1).

### 1.2. Aspartate Is a Nodal Metabolite

**Figure 2:**
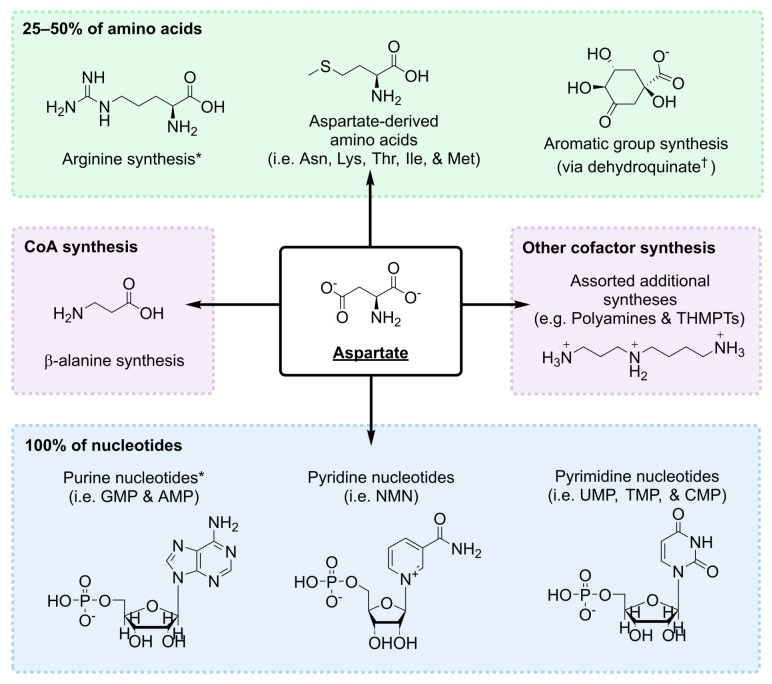
Aspartate occupies a nodal position in metabolism. It serves as a starting material or as a major synthetic component for an array of key metabolites across all extant metabolisms as well as a role as one of the proteinogenic amino acids. This graphic illustrates these major synthetic contributions. The green box indicates the contribution of aspartate to amino acid syntheses. The percentage indicates the proportion of the 20 proteinogenic amino acid repertoire with aspartate dependence; a range is presented as this is dependent on organism-specific metabolism. The blue box indicates aspartates contribution to nucleotide syntheses. The purple box indicates cofactors with aspartate dependence; CoA has is universal so is separated from other, organism-specific, cofactors. * Denotes synthetic process which utilise aspartate as a specialist amino group donor only. † Denotes synthetic process which are restricted to archaeal clades.

Of all amino acids in modern metabolism, aspartate is perhaps the most important. Aspartate does not have any unique chemistry for such a position, but rather occupies a nodal position in metabolism where it is used for the synthesis of many key species (Figure 2). In autotrophic prokaryotic organisms, between 25–50% of amino acids are derived from aspartate—Asn, Lys, Thr, Met and Ile, can all be directly synthesised from aspartate [36,37]. In archaea, aspartate participates in dehydroquinate synthesis [38], the first committed step of metabolism leading to the formation of most aromatic groups, including that of Trp, Phe and Tyr [39]. Aspartate also participates as an amino donor in the synthesis of arginine in bacterial, archaeal and eukaryotic metabolisms [40,41,42].

Clearly, this contribution to amino acid metabolism is important, but even more strikingly, aspartate is needed for the synthesis of all nucleotide species. It is the starting molecule for the synthesis of pyrimidine nucleotides. Much like in arginine biosynthesis, aspartate participates as an amino donor in the synthesis of inosine monophosphate (IMP), the precursor of all purines, and again in the synthesis of adenosine monophosphate (AMP) [43,44]. By extension, purine-derived species, e.g., thiamine-pyrophosphate, histidine, flavins, pterins and folates, are all linked to aspartate metabolism. It is also required across all prokaryotic metabolisms for the synthesis of nicotinamide mononucleotide (NMN) [45,46,47,48], one of the most important cofactors in most origins of life contexts [8,49].

Aspartate is frequently found in other cofactor syntheses, albeit with a more varied distribution across prokaryotes. For example, it is used for polyamine [50] and tetrahydromethanopterin synthesis [51] in some clades. However, aspartate is the primary route to the synthesis of β-alanine in most prokaryotic species [52,53]—via a pyruvoyl- [54,55] or pyridoxal- [56] catalysed mechanism. β-alanine is a universal component of coenzyme A (CoA) [57,58], a thiol species widespread in metabolism as a carboxylic acid-activating group [59].

### 1.3. Prebiotic Synthesis of Aspartate

In life, amino acids are synthesised by three mechanisms; the first mechanism involves the modification of an existing amino acid, such as the modification of aspartate to Asn, Thr, and Met. The second route is the direct reductive amination of α-keto acids by ammonia and a reducing H_2_ equivalent. In biological systems, this is almost exclusive to glutamate synthesis and represents the primary route of ammonia assimilation in biological systems [60]. In prebiotic experiments, Huber and Wächtershäuser [61] used FeS/(FeOH)_2_ minerals to reductively aminate various α-keto acids. Likewise, Barge et al. [62] used iron oxyhydroxides and pH gradients to drive alanine synthesis. Reductive amination of glyoxylate and oxaloacetate using FeS/Fe^0^ minerals generated with electrochemical methods has also achieved good yields of glycine and aspartate, respectively [63].

The third mechanism of amino acid synthesis in biology involves transamination between an amino acid species and a different α-keto acid. This mechanism is the primary way in which amino groups are distributed throughout metabolism. Non-enzymatic amino group transfer has been achieved multiple times in experimental systems [64,65,66]. Most recently, Mayer et al. [21] note that Cu^2+^ facilitates this transfer most readily. However, the transfer of amino groups in biological systems is rarely achieved via a direct mechanism; more often, the reaction is catalysed by pyridoxal-5-phosphate, a universally conserved and functionally diverse cofactor that is involved in up to 4% of modern enzymatic reactions [67]. Pyridoxal forms Schiff-base complexes with donor amino acids, before shifting to its pyridoxamine state; pyridoxamine then transfers its amino group to an acceptor α-keto acid [68] (Figure S1 contains an illustration of the pyridoxal–pyridoxamine transamination cycle). This reaction, and additional pyridoxal-dependent reactions, have been identified in enzyme-free systems [69,70,71].

All of this forms a body of evidence that amino acid syntheses can take place through multiple biomimetic chemical processes on the early Earth. The literature presents an issue in that biomimetic syntheses of aspartate are surprisingly under-represented. This is likely due to the poor stability of the α-keto acid precursor, oxaloacetate [72,73]. The only syntheses that start from oxaloacetate to date are those in the work of Kitadai et al. [63], which utilised electrochemically modified FeS minerals, the work of Krishnamurthy [74], which achieved synthesis using cyanide and diamidophosphate, and the work of Mayer and Moran [75], which used the reducing agent cyanoborohydride. None of these exactly mimic the biological synthesis route. Given the central importance of aspartate in metabolism, the limited evidence for its prebiotic synthesis is a problem for the protometabolism hypothesis. Because aspartate synthesis in biological systems utilises pyridoxal as a catalyst, this reaction may be the most prebiotically relevant process to explore. We set out to identify whether pyridoxal catalysis may be sufficiently fast to enable the synthesis of aspartate from oxaloacetate despite the limited stability of oxaloacetate. In addition, we hoped to define boundary conditions under which aspartate synthesis may be possible via this biomimetic protometabolic reaction.

## 2. Materials and Methods

### 2.1. Reagents

Aluminium chloride (99%), ammonium bicarbonate (≥99.5%), L-aspartic acid disodium salt (≥98%), L-alanine (≥99.5), β-alanine (≥99%), calcium chloride dihydrate (≥99%), chloroform (100–200 ppm amylene stabiliser, ≥99.5), cobalt chloride hexahydrate (cell culture grade), glycine (≥99%), glutamate (≥99%), guanidine chloride solution (8 M), hydrochloric acid (≥37%), isobutyl chloroformate (98%), manganese sulfate (cell culture grade), oxaloacetic acid (≥97%), potassium chloride (99%), potassium phosphate monobasic (99.5–101.0%), pyridine (98%), pyridoxamine dihydrochloride (cell culture grade), pyridoxal hydrochloride (≥99%), L-serine (≥99%), sodium acetate (99–101%), sodium orthovanadate (99.98%), and zinc chloride x (≥98%) were purchased from Sigma. Boric acid (analytical grade), water (HPLC grade) and acetonitrile (HPLC grade) were purchased from Fischer. Copper sulfate hexahydrate (98.0–102.0%), isobutanol (99%), magnesium chloride hexahydrate (≥98%), and nickel chloride hexahydrate (98%) were from Alfa Aesar. Sodium hydroxide was purchased from VWR, and sodium chloride (research-grade) was purchased from Millipore. Ammonium chloride (analysis-grade) was from Acros and 9-fluroenylmethoxycarbonyl chloride (97%) was from Novabiochem. Helium (99.995%) and Nitrogen (99.998%) were purchased from BOC.

### 2.2. General Reaction Conditions

Amounts of 10 mM oxaloacetate and 10 mM pyridoxamine hydrochloride were dissolved in 50 mM ammonium bicarbonate. The pH of the buffer solution was either left at a natural pH (7.8) or was adjusted using 3 M HCl or 5 M NaOH as appropriate. Reactant mixtures were then transferred to 15 mL falcon tubes and placed in an aluminium heat block (SciQuip) and, where appropriate, a sufficient volume of a 1 M metal catalyst stock was added immediately. The heat block was preheated to the appropriate temperature prior to transfer. Samples were taken at the time points listed in the figure caption and either directly derivatised or frozen at −20 °C prior to HPLC or GC-MS analysis.

### 2.3. Ionic Strength Screens

Sodium chloride solutions of appropriate strength or an artificial sea water mix, adapted from Sogin et al. [76], containing 26.37 g of L^−1^ NaCl, 5.67 g of L^−1^ MgCl_2_·6H_2_O, 1.47 g L^−1^ of CaCl_2_·2H_2_O, and 0.6 g of L^−1^ KCl were prepared. In each solution, 50 mM ammonium bicarbonate was dissolved and the pH was adjusted to 7.8. Reactions containing 5 mM oxaloacetate, 5 mM pyridoxamine and 1 mM CuSO_4_ were then prepared and moved to heat blocks.

### 2.4. Pressure Screen

Reaction mixtures containing 5 mM oxaloacetate, 5 mM pyridoxamine hydrochloride and 1 mM CuSO_4_ were prepared in 50 mM ammonium bicarbonate (pH 7.8) on ice. The solutions were moved to 25 mL glass headspace vials, also on ice, and crimped. Vials were pierced with 0.5 mm gauge needles and moved to a pressure reactor (Parr) with an open headspace vial containing HPLC-grade water. The reactor was assembled with the temperature insert in the open water vial. The reactor was placed on a hotplate with a thermostat and pressurised to 100 bars with nitrogen gas.

### 2.5. β-Alanine Synthesis

Amounts of 10 mM aspartate disodium salt and 10 mM pyridoxal were prepared in 50 mM ammonium bicarbonate (pH 7.8), and an appropriate volume of 1 M metal salt was then added before moving solutions to heat blocks.

### 2.6. Complete Reactions

Amounts of 5 mM donor amino acid, 5 mM oxaloacetate, 0.5 mM CuSO_4_ and either 1 or 5 mM pyridoxal was dissolved in 50 mM ammonium bicarbonate (pH 7.8) as required.

### 2.7. 9-Fluorenylmethylchloroformate (FMOC) Derivatisation

An amount of 100 µL of the sample was mixed with 200 µL of a 200 mM boric acid buffer (pH 9.5). An amount of 200 µL of 5 mM FMOC-Cl dissolved in acetonitrile was then added to this mixture and vortexed briefly. The sample was allowed to sit for 5 min at room temperature before being diluted 10-fold in a 40 mM phosphate buffer (pH 4.5). This diluted solution was passed through a 0.22 µm filter prior to HPLC analysis. This method was adapted from Jámbor and Molnár-Perl [77].

### 2.8. High-Performance Liquid Chromatography (HPLC) Analysis

Chromatographic separations took place on an Agilent Infinity II 1260 HPLC system with fluorescence and absorbance detectors. The column used was a Poroshell EC-C18 (4.6 mm × 150 mm, 4 µm) +5 mm guard column. The method employed a 1 mL/min flow rate using a gradient outlined in Table 1. Mobile phase A consisted of 50 mM sodium acetate at pH 4.5, mobile phase B was acetonitrile. The injections volumes were 1 µL with fluorescence detection at 266 nm for excitation and 320 nm for detection. Calibration curves are presented in Figure S2. Peak areas were determined via manual integration with the Chemstation software version 1.7 from Agilent technologies Wokingham, UK.

### 2.9. Isobutylchloroformate (iBuCF) Derivatisation

This derivatisation method was an adaptation of the method used by Wang et al. in 1994 [78]. An amount of 100 µL of the samples, 50 µL of 5 M NaOH and 100 µL isobutanol:pyridine (3:1) were vortexed together in 1.5 mL Eppendorfs. Immediately after vortexing, 50 µL of iBuCF was added and mixed by pipetting up and down rapidly. This was then capped and vortexed, and the Eppendorf was opened afterwards to release any residual pressure build-up. Derivatised samples were then extracted via an addition of 100 µL of chloroform. Eppendorfs were vortexed vigorously before transferring the organic layer to 100 µL inserts for GC-MS analysis. Calibration curves are given in Figure S3.

### 2.10. Gas Chromatography Mass Spectrometry (GC-MS) Analysis

GC-MS analyses were carried out on Agilent 6890 GC with 5975 MSD. Separation was achieved using Agilent ZORBAX DB5-MS (40 m × 250 µm × 0.25 µm) using helium as the carrier gas with a constant flow at 1.1 mL/min passing through a PSD plate and then through an additional column (deactivated fused silica, 2 m × 250 µm) to the GC-MS instrument. An amount of 1 µL of the sample was introduced in the splitless mode. The front inlet temperature was maintained at 250 °C. The initial oven temperature was 60 °C and held for 1 min, and the temperature was then increased at a rate of 10 °C/min to 280 °C and held for 10 min. The MSD transfer line and source temperatures were 290 °C and 250 °C, respectively.

Derivatised standards of amino acids were analysed in scan mode (50–300 *m*/*z* units) to obtain reference spectra and retention times. Fragment ions were confirmed manually using ChemDraw. Primary ions and qualifier ions were selected from these spectra and used to build a selected ion monitoring (SIM) method—see Table 2 for the list of ions and their relative intensities used. All dwell times were 50 ms. Peak deconvolution was performed by the MassHunter quantitative analysis package from Agilent.

## 3. Results

### 3.1. Transamination of Oxaloacetate by Pyridoxamine

#### 3.1.1. Effect of Transition Metal Ions

**Figure 3:**
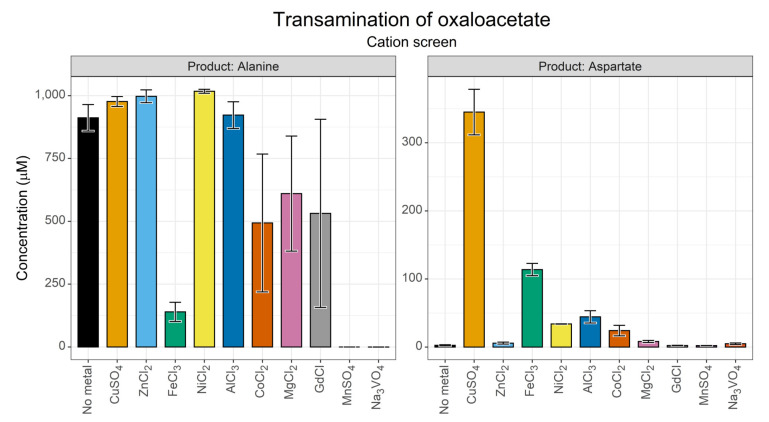
Results of a cation screen for transamination of oxaloacetate. Yields of alanine and aspartate after 24 h, as determined using the FMOC derivatisation and HPLC separation, when 10 mM oxaloacetate and 10 mM pyridoxamine were incubated at 70 °C in 50 mM a ammonium bicarbonate buffer (pH 7.8), with a 1 mM cation catalyst. *N* = 3 ± SD.

The activity of pyridoxal species is known to be influenced by transition metals, with which they form complexes [66]. In prebiotic contexts, metal ions are widely agreed to be one of the most plausible catalysts in a wide range of origins scenarios [13,32]. For these reasons, they were the initial factor that we chose to screen. We selected a range of transition metals common to biological systems or plausible in terms of geochemistry and included guanidine as an analogue for arginine in enzyme structures. This initial screen (Figure 3) was conducted at 70 °C, as we hoped to identify conditions under which aspartate could be generated despite the instability of oxaloacetate that was expected at this temperature.

Synthesis of both alanine and aspartate appeared to take place in most reactions with chromatographic peaks above baseline levels. Curiously, Fe^3+^ appeared to suppress alanine yield compared to equivalent reactions. The aspartate yield in the control reaction was 2.6 µM, a 0.026% conversion and an alanine yield of ~911 µM corresponding to a 9.1% conversion. In fact, the yield of alanine was likely to be even higher than that, as the FMOC method was optimised for low concentrations (1–500 µM range); above this range, there is likely to be detector saturation, as evidenced by the flat tops of the alanine peak (Figure S4). Cu^2+^ ions showed the greatest aspartate yield (3.45%), which is in line with previous studies on transamination reactions [21]. However, several of the cations screened showed a substantial aspartate yield including that of Ni^2+^ (0.34%), Al^3+^ (0.44%), and Fe^3+^ (1.33%).

Overall, these data suggest that the transamination of oxaloacetate by pyridoxamine can take place under non-optimal temperature conditions, when aided by a range of transition metal ions. Cu^2+^ clearly showed the best rate enhancement. Interestingly, Mn^2+^ and VO_4_^3-^ ions showed negligible synthesis of either alanine or aspartate, perhaps reflecting a suppression of transamination activity.

#### 3.1.2. GC-MS Secondary Verification

**Figure 4:**
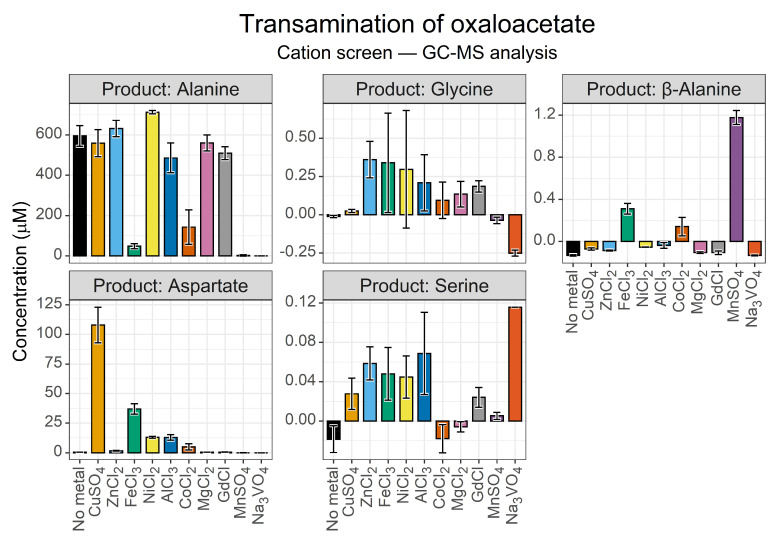
GC-MS secondary verification of cation effects on oxaloacetate transamination. Yields of selected amino acids after 24 h, as determined via iBuCF derivatisation and GC-MS separation, when 10 mM oxaloacetate and 10 mM pyridoxamine were incubated at 70 °C in a 50 mM ammonium bicarbonate buffer (pH 7.8), with a 1 mM cation catalyst. Sample concentrations were normalised relative to a water sample. Data are secondary verifications of the HPLC data in Figure 3. *N* = 3 ± SD.

HPLC verification was our primary analytical technique. Secondary verification, however, is required to definitively prove the synthesis of any metabolite, as false positives are possible. We opted to use iBuCF derivatisation with GC-MS analysis as the combination of retention time and fragment ion patterns provide high reliability with minimal preparation. The derivatisation method works for amino species in aqueous solution, so it avoids any artifacts introduced through drying. We tracked the formation of selected amino acid species of greatest interest in relation to aspartate synthesis—alanine, β-alanine, glycine and serine are some of the closest amino acids within metabolism (Figure 4).

While the metal screen showed a reduced aspartate yield compared to HPLC analysis, the external standard also showed a reduced signal, so this is likely due to analytical complications. The GC-MS analysis was intended to confirm synthesis, not for precise yield analysis. In that light, our analysis confirmed the formation of aspartate and alanine with the same optimal synthesis of aspartate in the presence of Cu^2+^ ions. The GC-MS method also confirmed the unusually low levels of alanine in the reaction catalysed by Fe^3+^. This experiment indicated that the yield of alanine in the presence of Co^2+^ ions could be overestimated in the HPLC data and confirmed that neither alanine nor aspartate synthesis took place in the presence of Mn^2+^ and VO_4_^3-^ ions. However, given that both our derivatisation procedures for HPLC and GC-MS employed chloroformate species, it is possible that these ions interfered with this analysis.

The GC-MS analysis also revealed a small β-alanine peak present in the Fe^3+^, Cu^2+^ and Mn^2+^ samples (Figure 4, panel 3). β-Alanine levels were extremely low, with 1.2 µM being the maximal yield in the Mn^2+^-catalysed reaction, and 0.31 µM and 0.14 µM being the maximal yield in the Fe^3+^- and Co^2+^-catalysed reactions, respectively. Prokaryotes can synthesise β-alanine through the decarboxylation of aspartate by either a pyruvoyl group [54], or by pyridoxal-5-phosphate cofactors [53], both of which can be found in this reaction system—pyridoxal from the amino group transfer from pyridoxamine, and pyruvate from the degradation of oxaloacetate. However, β-alanine synthesis did not scale with aspartate synthesis, indicating different catalytic requirements. Alternatively, this may indicate that oxaloacetate undergoes preceding oxidative decarboxylation to 3-oxopropanoic acid, which in turn reacts with pyridoxamine to form β-alanine. Such a reaction would mimic a reversed β-alanine degradation pathway [79].

#### 3.1.3. PH Screen

One question we wanted to explore in this investigation was the environmental conditions that enable protometabolic transamination reactions to take place. This context can be used to define both the optimal conditions and the boundary limits of the reaction, which together help frame the environmental parameters that permit a non-enzymatic protometabolism to take place. One of the principal conditions we wanted to explore was the pH of the reaction system. Using CuSO_4_ as the metal catalyst, a trio of reactions across a range of pH 6–10 were conducted. We did not explore strongly acidic conditions.

**Figure 5:**
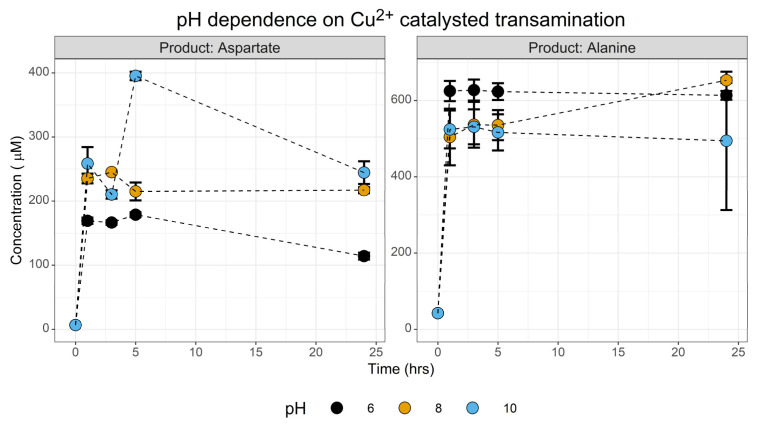
Investigating the effect of pH on the transamination of oxaloacetate. Yields of aspartate and alanine were determined during a 24 h time course across three different pHs via the FMOC-HPLC method. Reactions were conducted with 10 mM pyridoxamine and 10 mM oxaloacetate in 50 mM ammonium bicarbonate with 1 mM CuSO4 at 70 °C. *N* = 3 ± SD.

This screen (Figure 5) gave two primary insights. First, synthesis of both aspartate and alanine took place at all three pH conditions screened. Statistical analysis at t = 1 hr shows that for aspartate, pH had a statistically significant effect between the acidic and alkaline pH (one-way ANOVA, F = 17.3, *p* = 0.003. TukeyHSD: pH 8 ~ pH 6, *p* = 0.01; pH 10 ~ pH 6, *p* = 0.003; pH 10 ~ pH 8 *p* = 0.36). The reciprocal test for a pH effect on alanine production was not significant. This reflects either the pK_a_ of the amino group which needs to be deprotonated to act as a nucleophile required for reaction with the ketone of oxaloacetate, or a modest stabilisation of oxaloacetate taking place at alkaline pH. The second observation is that the reaction is extremely fast, with a ~160–258 µM yield within the first hour. This indicates that Cu^2+^-catalysed transamination probably out-competes the decarboxylation of oxaloacetate in this initial stage of the reaction.

#### 3.1.4. Temperature Screen

**Figure 6:**
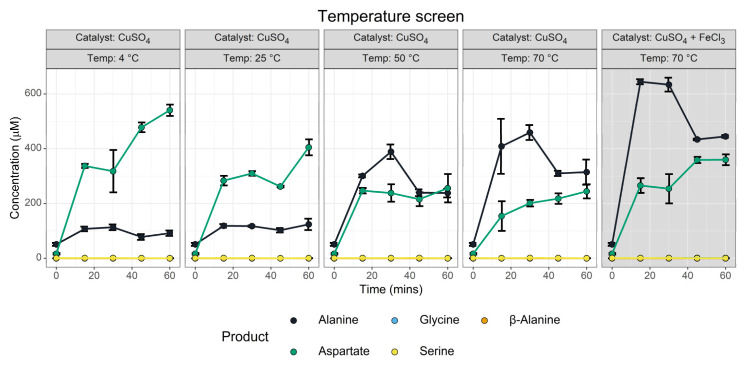
Effect of temperature on transamination of oxaloacetate. Yields of amino acids determined during a 1 h reaction across five different conditions via the iBuCF GC-MS method. Reactions were conducted with 10 mM pyridoxamine and 10 mM oxaloacetate in 50 mM ammonium bicarbonate (pH 7.8) with 1 mM CuSO_4_ (Panel 5, in grey, with additional 1 mM FeCl_3_) as required. T = 0 is common for all temperature screens. *N* = 3 ± SD. All values have been normalised relative to a water blank. Yields of glycine, serine and β-Alanine are negligible but further details are presented in Figure S7.

We anticipated that temperature would be a major determining element, as the stability of oxaloacetate is known to heavily depend on temperature. In parallel, we screened a reaction containing both Cu^2+^ and Fe^3+^ ions (Figure 6, grey panel), which was intended to explore whether or not the low alanine yield in the cation screen reflected a stabilisation of oxaloacetate by Fe^3+^ ions.

The temperature screen (Figure 6) identified that aspartate synthesis took place across all temperatures, though higher temperatures had lower aspartate yields. We infer that the transamination reaction was so fast that it is nearly complete before the solutions could fully equilibrate with the heat block, rather than there being an unexpected stabilisation of oxaloacetate. This inference is supported by the non-zero yields of aspartate and alanine present at the t = 0 time point, which was further complicated by the time for the derivatisation procedure and thawing that were part of the analysis. In contrast, the alanine yield showed substantial temperature dependence. This was not unexpected, given that the thermal decomposition of oxaloacetate liberates pyruvate, which can be transaminated. No serine nor β-alanine synthesis could be observed, though minor glycine synthesis could be observed (Figure S6).

Fe^3+^ and Cu^2+^ may have cooperative effects. The GC-MS data (Figure 6, panels 4 and 5) show increased yields of both alanine and aspartate. The HPLC data do not show any significant difference for aspartate but do for alanine (Figure S7). This suggests that Fe^3+^ may have a stabilising effect on oxaloacetate; however, further experimentation is needed for definitive confirmation as increased yields may simply reflect increased transamination activity due to greater transition metal ion abundance.

#### 3.1.5. Ionic Strength

Ionic strength is known to effect reductive amination reactions [61] and is one of the primary characteristics that differentiate between scenario-driven origins of life hypotheses. We set up a series of reactions between pyridoxamine and oxaloacetate in increasing-strength NaCl solutions and in a simplified ‘ocean ion’ mixture [76]. This ocean mixture was not intended to represent Archaean ocean conditions, but rather to indicate whether or not more complex salt mixtures change outcomes.

**Figure 7:**
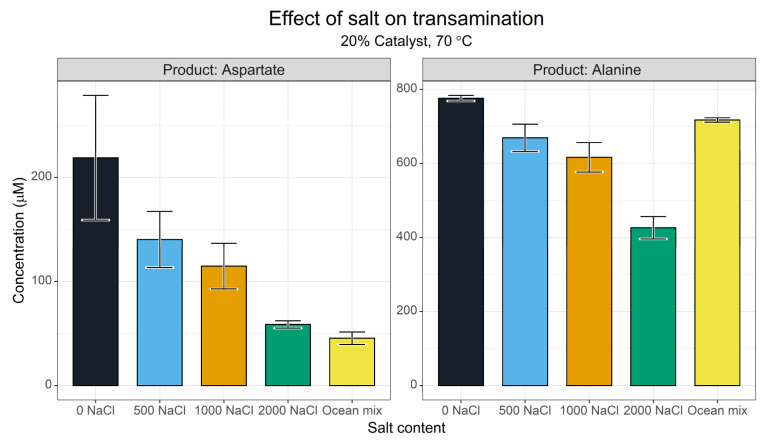
Effect of ionic strength on the transamination of oxaloacetate. Yields of aspartate and alanine after a 3 h reaction as determined via the FMOC-HPLC method. Reactions were conducted with 5 mM pyridoxamine and 5 mM oxaloacetate in 50 mM ammonium bicarbonate (pH 7.8) at 70 °C with 1 mM CuSO_4_, and the appropriate salt mixture as required. The unit of NaCl is mM. *N* = 3 ± SD.

The screen (Figure 7) showed that increasing NaCl content inhibited both aspartate and alanine synthesis, presumably by suppressing the transamination reaction. It should be noted that even in the 2 M NaCl reaction, the synthesis of neither amino acid was completely suppressed. The amino acid concentrations plot linearly with added NaCl. The ocean mixture (which contains 450 µM NaCl), deviated, however—in this case, the aspartate yield was disproportionately low relative to the ionic strength, whereas alanine yield was improved. The ocean mix contained MgCl_2_ and CaCl_2_ which may have competed with Cu^2+^ to complex with the pyridoxamine-altering transamination.

Interestingly, GC-MS secondary verification (Figure S8) showed a more modest inhibition of aspartate synthesis. Comparing the 2000 mM NaCl to the 0 mM NaCl experiment, the HPLC data showed a 73% reduction in aspartate yield whereas GC-MS only gave a 36% reduction. This may reflect additional ionic strength effects operating on the derivatisation processes. Both data sets show a suppression in transamination with salt, however. In addition, the ocean mixture had a low but significant effect on the synthesis of glycine compared with NaCl alone. This suggests that the effects of MgCl_2_ and CaCl_2_ on the system may be more complex than expected.

#### 3.1.6. Pressure Effects

Pressure can vary greatly between different origins of life environments, with deep-sea hydrothermal vents having the greatest pressure condition of current theories [80,81,82,83].

For aqueous reactions, it is typically taught that pressure does not affect the reaction equilibrium. This is a slight misconception, however, as hydrostatic pressure can influence equilibrium due to differences in the aqueous volume of reactants and products, an extension of le Chatelier’s principle [84]. In the context of this reaction, the decomposition of oxaloacetate generates carbon dioxide, which in turn will partition out of solution according to Henry’s law. Higher pressures may offset both processes, shifting the equilibrium of the reaction.

**Figure 8:**
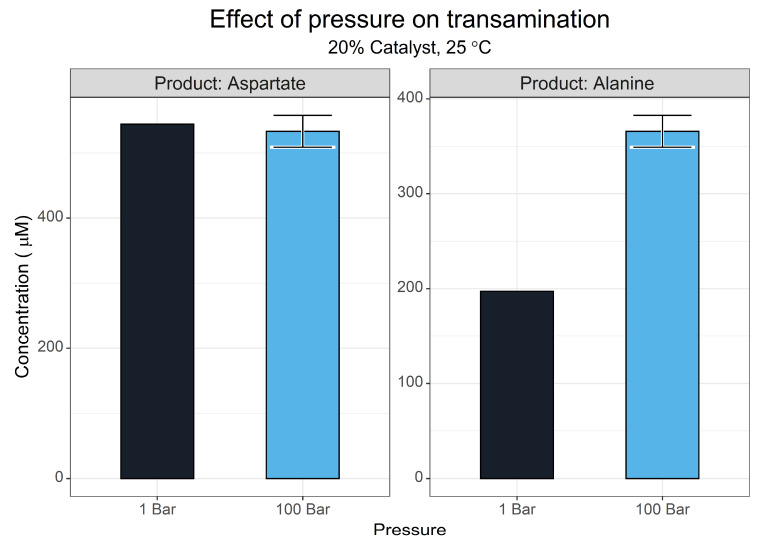
The effect of pressure on the transamination of oxaloacetate. Yields of aspartate and alanine after a 3 h reaction under pressure as determined via the FMOC-HPLC method. Reactions were conducted with 5 mM pyridoxamine and 5 mM oxaloacetate in 50 mM ammonium bicarbonate (pH 7.8) at 25 °C with 1 mM CuSO_4_. *N* = 3 ± SD.

Due to the limitations of the pressure reactor being used, a single reaction was set up with triplicate reactions. A single atmospheric control was set up in parallel. Figure 8 shows that aspartate synthesis did not change between the two conditions. In contrast, alanine yield showed an increase of 168 µM with a 100-bar pressure. This might have been due to the change in temperature when the pressure of the system was increased, or it may have been that pressure increased total transamination activity by shifting the position of the pyruvate–alanine equilibrium.

### 3.2. β-Alanine Synthesis

The synthesis of β-alanine in our initial metal screen was unexpected, and raised questions about how this reaction was taking place. We imagined that different metals may have adjusted the catalytic nature of pyridoxal, which is known to have multiple catalytic functions [67]. In fact, non-enzymatic decarboxylation reactions catalysed by pyridoxal have been reported [71]. We decided to investigate the formation of β-alanine from aspartate with a limited temperature and metal ion screen. We decided to use Fe^3+^ instead of Mn^2+^, as Fe^3+^ enabled transamination activity for both aspartate and alanine, whereas Mn^2+^ by contrast suppressed all transamination activity.

**Figure 9:**
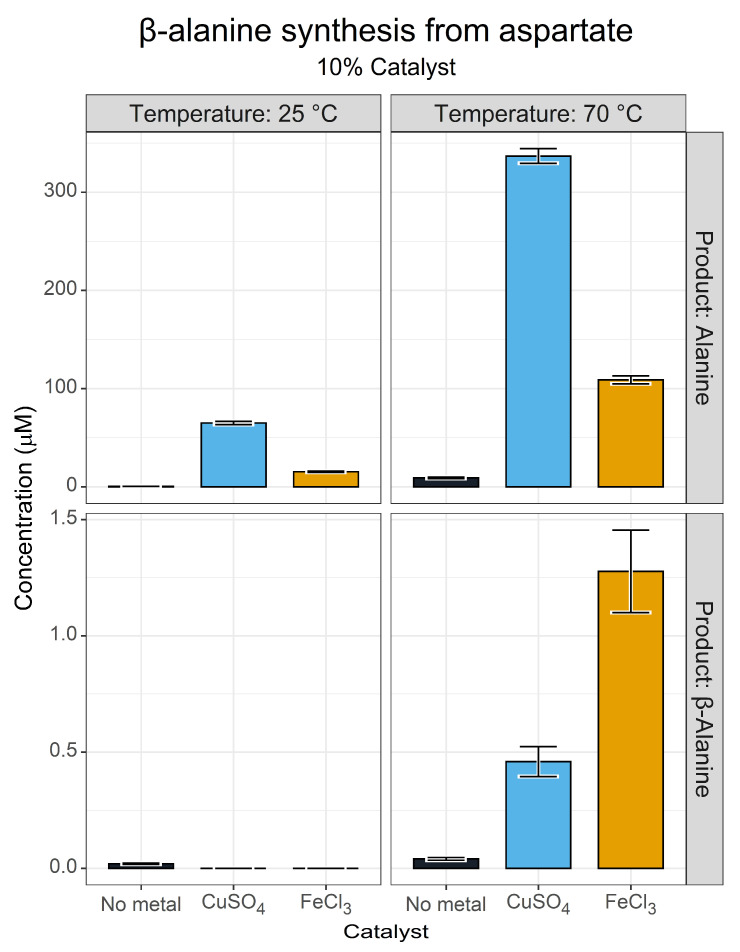
β-alanine synthesis from aspartate. Yields of alanine and β-alanine after a 3 h reaction as determined via the FMOC-HPLC methods. Reactions contained 10 mM aspartate and 10 mM pyridoxal in 50 mM ammonium bicarbonate (pH 7.8) with a 1 mM metal catalyst. *N* = 3 ± SD.

Figure 9 shows that β-alanine exhibits a catalytic and temperature dependence with little to no production at 25 °C or at 70 °C in the absence of a metal catalyst. At 70 °C, however, yields of β-alanine of between 1 µM (HPLC) and 6 µM (GC-MS) were obtained with Fe^3+^ catalysis. This may indicate that the decarboxylation reaction had a higher energetic requirement. Our GC-MS analysis only confidently identified β-alanine in the Fe^3+^ reactions but did not indicate yields above the control in the Cu^2+^-catalysed reaction. Interestingly, we also found an indication of very minor glycine synthesis in the same reaction (Figure S9). This may reflect an alternative deacetylation reaction, or a more complex breakdown network having taken place. 

This experimental system also indicated that a complete pyridoxal cycle was taking place. The generation of alanine in this reaction system relied on the formation of oxaloacetate generated by the deamination of aspartate. Decarboxylation of this oxaloacetate generated pyruvate, which in turn was transaminated to alanine. This sort of catalytic cycle is exactly the behaviour expected for pyridoxal in protometabolic and biological contexts.

### 3.3. Complete Pyridoxal Cycles for the Synthesis of Aspartate

The evidence of a complete cycle taking place between aspartate and pyridoxal to generate alanine prompted a final investigation to attempt the synthesis of aspartate, using alanine as a donor (reversing the previously described reaction).

**Figure 10:**
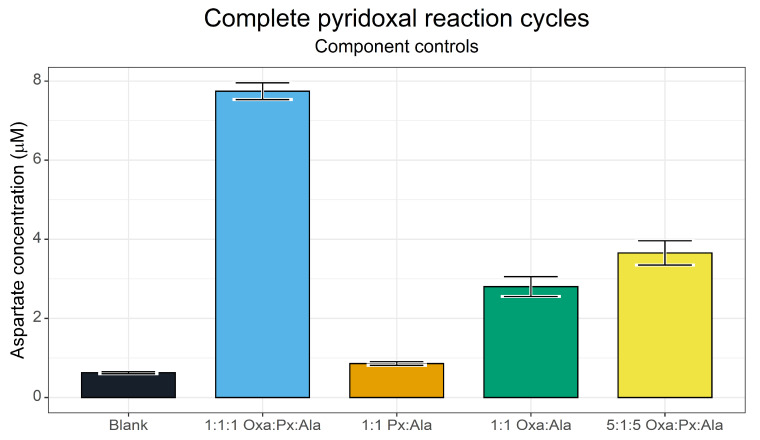
Exploring amino group transfer from alanine to oxaloacetate using pyridoxal. Yields of aspartate after a 3 h reaction as determined via the FMOC-HPLC method. Ratios and component mixtures are given for each reaction. Oxa = oxaloacetate, Px = pyridoxal, Ala = alanine. The maximal concentration for any species is 5 mM. Reactions were conducted at 70 °C in 50 mM ammonium bicarbonate (pH 7.8). *N* = 3 ± SD.

An initial investigation (Figure 10) at 70 °C focused on the component controls, analysing the contribution of pyridoxal to the transamination reaction. We screened a complete reaction mixture (1:1:1 oxaloacetate (Oxa):pyridoxal (Px):alanine (Ala), with all reactants at 5 mM, and 0.5 mM CuSO_4_), without oxaloacetate (1:1, Px:Ala), without pyridoxal (1:1, Oxa:Ala) and a reaction with a reduced pyridoxal content to assess the catalytic power of pyridoxal (5:1:5 Oxa:Px:Ala, 1 mM pyridoxal).

This screen identified that holistic cycling of pyridoxal could indeed take place. However, the yield of aspartate was considerably lower than that achieved via direct transamination by pyridoxamine. This indicates that, whilst the transamination directly from pyridoxamine was fast enough to outcompete the decarboxylation reaction, the entire catalytic cycle was much slower. The component controls showed that a basal level of direct amino transfer was possible between alanine and oxaloacetate, probably through a similar mechanism to that described by Moran [21]. The lower concentration of pyridoxal showed a small improvement over this basal activity.

**Figure 11:**
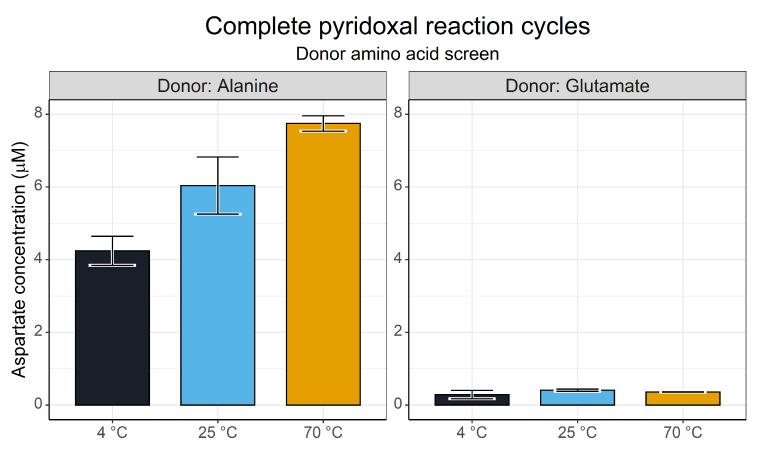
Yields of aspartate after a 3 h reaction as determined via the FMOC-HPLC method. Reactions contained 5 mM donor amino acid species, 5 mM pyridoxal and 5 mM oxaloacetate in 50 mM ammonium bicarbonate (pH 7.8) with a 1 mM CuSO_4_ catalyst at 70 °C. *N* = 3 ± SD.

We conducted a screen across several temperatures and with different amino group donors (alanine and glutamate are, along with aspartate, the primary amino group donors in metabolism [60]) to test whether the aspartate yield varied with different donors. This simple 3 h screen (Figure 11) showed a startling distinction between the two donor amino acids. Alanine generated notable yields of aspartate with a clear temperature dependence, likely reflecting the rate requirements for the two reaction steps. Glutamate by contrast showed little to no aspartate synthesis.

## 4. Discussion

### 4.1. Copper-Assisted Transamination Outcompetes Decarboxylation

The combined data demonstrate that aspartate synthesis could take place from oxaloacetate provided that the transamination reaction was catalysed fast enough to outcompete the degradation of oxaloacetate. Aspartate synthesis was observed in almost all reactions, with substantial yields within 15 min of the reaction across temperature ranges. The Cu^2+^–pyridoxamine transamination operated with a maximum yield of ~5% (4 °C, 1 h) though run-to-run and analytical variation were observed. Some of the variation is likely reflected by the nature of the analytical set-up. Specifically, the degradation of oxaloacetate in prepared solutions, the rapid kinetics of Cu^2+^-catalysed transamination, the derivatisation times and the time taken to thaw samples all contributed to this variation. This may also account for the differences between GC-MS and HPLC techniques. Whilst the exact yields may be debatable, the overall trends that we observed are repeatable and have been confirmed by both methods.

One factor that has not been investigated here, but which should be in future investigations, is the effect on the pyridoxal catalyst. The reactions, particularly those undertaken at higher temperatures, can undergo substantial colour changes. These colour changes are metal ion dependent. For example, Cu^2+^ generates an orange colour, while Fe^3+^ generates a deep red colour. Colour development takes place over time and is temperature-dependent, so we believe this reflects the degradation of the pyridoxal species rather than that of different metal ion–pyridoxal–amino acid complexes. If so, this has obvious implications for the prebiotic plausibility and suitability of these metal ions in a protometabolic network. 

### 4.2. Environmental Boundary Conditions

The environmental conditions explored here offer a limited window into the wide range of possible conditions that encompass all origins-of-life settings. The metal screen showed that Cu^2+^ availability at the origins of life may have been an important factor (in line with several papers [22,75]), though we note that many biologically and geochemically relevant ions also facilitate the transamination. Much like in enzymatic contexts, transamination occurs in the absence of metal ions too. Given that metal ions are known catalysts for oxaloacetate decarboxylation, this is particularly relevant [85,86]. The contribution of the anions is unknown and should be considered in further experimental investigations.

We found that Cu^2+^-catalysed aspartate synthesis took place rapidly across a wide range of pH. There was a slight preference for alkaline conditions, which reflects either the deprotonation of pyridoxamine or a stabilisation of oxaloacetate. Pressure did not supress the transamination of oxaloacetate but may have had negative effects on the stability of oxaloacetate, as evidenced by increased alanine levels, implying that a high pressure may not be helpful. An alternative explanation could be that pressure increased the total transamination activity by shifting the keto-acid/amino acid equilibria. The yields of aspartate versus alanine show a temperature dependence that aligns with the stability of oxaloacetate, with the yield of aspartate being greatest at lower temperatures. However, caution should be taken when interpreting our temperature screen, as all the reactions were prepared on ice before being transferred to their aluminium heat blocks. Given the fast rate at which our transaminations took place, it is possible that the bulk solvent had not fully equilibrated with the temperature of the heat block. Nonetheless, the time course shows that transamination did occur at all temperatures surveyed here.

The ionic-strength screen indicates that the transamination of both oxaloacetate and pyruvate have an inverse relationship with ionic strength. This aligns with previous work by Huber and Wachterhauser [61]. The 2000 mM NaCl screen went well beyond the predicted ionic strength of the Hadean ocean [87,88], but does indicate that lower salt conditions would favour an optimal yield. There is no indication that the increased salt affected the solubility of CuSO_4_ or the pyridoxal species, which could in principle contribute to this effect. The artificial ocean salt mixture also showed a suppressed aspartate yield but an increased alanine level; these two effects in combination might indicate that the artificial ocean mixture increased oxaloacetate breakdown, but further work would be required to definitively prove this.

Overall, our screens show that transamination of oxaloacetate can take place across a wide range of conditions, provided of course that oxaloacetate is sufficiently available. For this reason, we believe that this reaction could have utility in a range of astrobiological and origin-of-life hypotheses, though exploration within specific environmental contexts is necessary to fully evaluate this [89]. The conditions explored indicate that maximal yields would be formed in a cooler, alkaline and low-ionic-strength environments with Cu^2+^ catalysis. Cu^2+^ is enriched in hydrothermal fluids compared to seawater in several modern hydrothermal systems [90] and may have been the case in prebiotic hydrothermal systems [81], though the environment may have to be low in sulphide given the low solubility of copper sulphides [91].

### 4.3. β-Alanine Synthesis at the Origins of Life

During this investigation, we identified that β-alanine synthesis could take place at very low levels (1 µM or a 0.01% yield in 24 h) when catalysed by Fe^3+^ ions and exhibited strong temperature dependence. This reaction reflects a non-enzymatic version of the aspartate decarboxylase from *Methanocaldococcus jannaschii* [53]. Further work may focus on identifying whether or not this reaction involved the participation of pyruvate derived from a reaction analogous to bacterial aspartate decarboxylase.

Such a low yield questions whether or not this reaction has meaningful prebiotic relevance, but resolving it is nuanced, beyond the scope of this paper and is perhaps better-suited to modelling approaches. Even so, it is worth emphasising that the primary role of β-alanine in metabolism is as a component of CoA. CoA is a widespread and essential cofactor involved in multiple metabolic pathways. As such, it is possible that even limited β-alanine synthesis could have significant autocatalytic effects on a protometabolic network [16].

Interestingly, glycine was synthesised at significant levels compared to controls. Yields never exceeded 1 µM. How glycine was synthesised from these reactions is unclear, but its presence indicates that the reaction network is likely more complex than we have described here.

### 4.4. Complete Pyridoxal/Pyridoxamine Cycles

In life, pyridoxamine is found only as a transition state in aminotransferase enzymes. Our results show that the complete cofactor cycle can in fact proceed from aspartate to alanine (Figure 9). However, we could not generate substantial yields of aspartate in the reverse direction, which may reflect several factors.

First, it could be that the two half-reactions of the pyridoxal cycle took place at different rates. If so, this would suggest that Cu^2+^ catalysis is amino acid-dependent, but that seems unlikely given earlier work [21]. Second, it is possible that the thermodynamics or kinetics of the system do not facilitate the reverse reaction. Oxaloacetate is unstable under conditions screened and degrades readily to release CO_2_. This in turn partitions out of solution under these reaction conditions, facilitating further decarboxylation of oxaloacetate. The CO_2_-saturated Hadean oceans might offset this effect, especially at higher pressures. In that case, changing the solvent conditions to a CO_2_-saturated solution may shed more light on this mechanism. Finally, we did not add pyruvate to the solution, so oxaloacetate was even further from equilibrium under the conditions studied, and hence was even more likely to break down. Coupling this transamination reaction to more complex protometabolic experiments may prove more fruitful. However, whatever the reason for the differences in rates, the fact that alanine was a better amino donor than glutamate was surprising, given the role of glutamate as the primary amino group donor in modern metabolism. This observation warrants further investigation.

### 4.5. Future Perspectives

The work presented in this paper focuses on a specific sub-reaction within a larger plausible metabolic network, specifically seeking to understand whether aspartate can be synthesised from oxaloacetate in simple, prebiotic contexts. In the wider protometabolic context, one of the major questions is the availability of oxaloacetate. No prebiotic synthesis of oxaloacetate, in an autotrophic direction, has been shown—though its availability can be inferred from more complex mixed direction systems [20]. The reaction presented here shows that if oxaloacetate can indeed be produced, then a sustained production of aspartate could take place through metal–pyridoxamine transamination, rapidly offsetting any degradation of oxaloacetate. In other words, as in modern systems, oxaloacetate was arguably never available in abundance, but rather was formed continuously and converted rapidly into the more stable nodal metabolite aspartate. This is an important aspect of the protometabolism hypothesis. Many metabolic intermediates are unstable and will never accumulate in the environment. There is no need for them to do so. What is required is that they are formed continuously via protometabolic flux, driven by far-from-equilibrium conditions in the environment—ultimately the disequilibrium between H_2_ and CO_2_, which are the dominant precursors of core autotrophic metabolism [80,81,92,93].

The other side of such a scenario is the requirement that pyridoxamine is available in sufficient quantities. The evidence from our complete reactions suggests that amino group transfer from any amino acid to oxaloacetate via pyridoxamine cannot produce much aspartate. This result does not preclude the possibility that pyridoxamine is produced in the system, but suggests that amino group transfer may have taken place too slowly to form aspartate before the oxaloacetate broke down. A reaction system where oxaloacetate is generated continuously, as noted above, may offset this problem. Likewise, the fact that pyridoxamine is never found outside an enzyme’s transition state in modern systems does not mean that pyridoxal and pyridoxamine could not exist as independent entities in equilibrium under prebiotic conditions. A direct reductive amination of pyridoxal might even be possible under such conditions.

The final missing element in such a scenario is the availability of pyridoxal. Pyridoxal is a unique cofactor in metabolism in that it does not explicitly require any amino acid for its synthesis. In most organisms, pyridoxal-5-phosphate is produced from glyceraldehyde-3-phosphate, ammonia, and ribose-5-phosphate in a single enzymatic process [94], the ammonia derived from glutamine hydrolysis in a separate enzyme. The exact prebiotic availability of these species is unknown, but pentose and triose sugars can be readily produced in the formose reaction [95,96,97]. The prebiotic relevance of the formose reaction is questionable, and is even more so in the context of protometabolism [95]. However, pyridine species such as pyridoxal can be generated from glycolaldehyde in ammonia solutions, suggesting that biomimetic synthesis is not implausible [98].

## 5. Conclusions

This work demonstrates that the synthesis of aspartate is possible in aqueous solution at low yields, following non-enzymatic, biomimetic synthesis. Our investigation is far from exhaustive, and only provides part of the answer to how aspartate became available in the context of the protometabolic origin of life. Nonetheless, we do address the critical underrepresentation of aspartate in protometabolic amino acid syntheses and point to further downstream reactions that mimic those of aspartate in modern metabolism, including the synthesis of β-alanine. These reactions depend largely on the availability of simple metal-ion catalysts and on one of the simplest and most deeply conserved cofactors. Our findings are consistent with the hypothesis that the nodal position of aspartate in modern metabolism was conserved from protometabolic reaction networks at the origin of life.

## Figures and Tables

**Table 1:** Gradient programme used for the HPLC separation of FMOC-derivatised amino acids.

Time (mins)	Mobile Phase A (%)	Mobile Phase B (%)
0.00	72	28
2.38	72	28
21.42	55	45
25.39	5	95
29.36	5	95
30.95	72	28
50.00	72	28

**Table 2:** Key variables used in the SIM analysis of iBuCF-derivatised amino acids detected via GC-MS. The table lists amino acids and their retention times, primary diagnostic masses and several qualifier masses and their relative intensities.

Amino Acid (Retention Time)	Primary Ion, *m*/*z*	Qualifier Ions (Relative Intensity %)
Alanine (15.49)	144.0	88.0 (20.2), 116.0 (11.3)
Glycine (15.69)	133.0	102.0 (32.4), 120.0 (18.0), 176.0 (17.6)
β-Alanine (16.66)	116.0	88.0 (38.1), 98.0 (37.6), 143 (45.4)
Aspartate (20.85)	244.0	88.0 (43.5), 144 (23.8), 160.0 (12.4)
Serine (21.52)	204.0	86.0 (90.9), 148.0 (64.2)

## Data Availability

The data that support the findings of this study are available upon request.

## Supplementary Materials

The following supporting information can be downloaded at: https://www.mdpi.com/article/10.3390/life13051177/s1, Figure S1: Schematic of the transamination reaction cycle. Figure S2: FMOC-Amino acid calibration curves. Figure S3: iBuCF derivatized amino acid calibration curves. Figure S4: Example HPLC chromatogram. Figure S5: GC-MS verification for pH screen. Figure S6: Temperature screen data in expanded format. Figure S7: HPLC data for the temperature screen. Figure S8: GC-MS verification of the ionic strength screen. Figure S9: GC-MS verification of β-alanine synthesis from aspartate.
