# Supplementary material for: Prebiotic Synthesis of Aspartate Using Life’s Metabolism as a Guide
Source: Life (Basel). 2023 May 12;13(5):1177. doi: 10.3390/life13051177 (PMC10221237; doi:10.3390/life13051177)
Supplement: Supplementary file 1 [file life-13-01177-s001.zip › life-2366299-supplementary.pdf]

Supplementary figures

Prebiotic Synthesis of Aspartate Using Life's Metabolism as a Guide

Stuart A. Harrison, William L. Webb, Hanadi Rammu and Nick Lane

Centre for Life's Origins and Evolution (CLOE), Department of Genetics, Evolution and Environment, University College London, Gower Street, London WC1E 6BT, UK

Correspondence: stuart.harrison.17@ucl.ac.uk or nick.lane@ucl.ac.uk

Key words: protometabolism, origins of life, aspartate, oxaloacetate, pyridoxal, metabolism

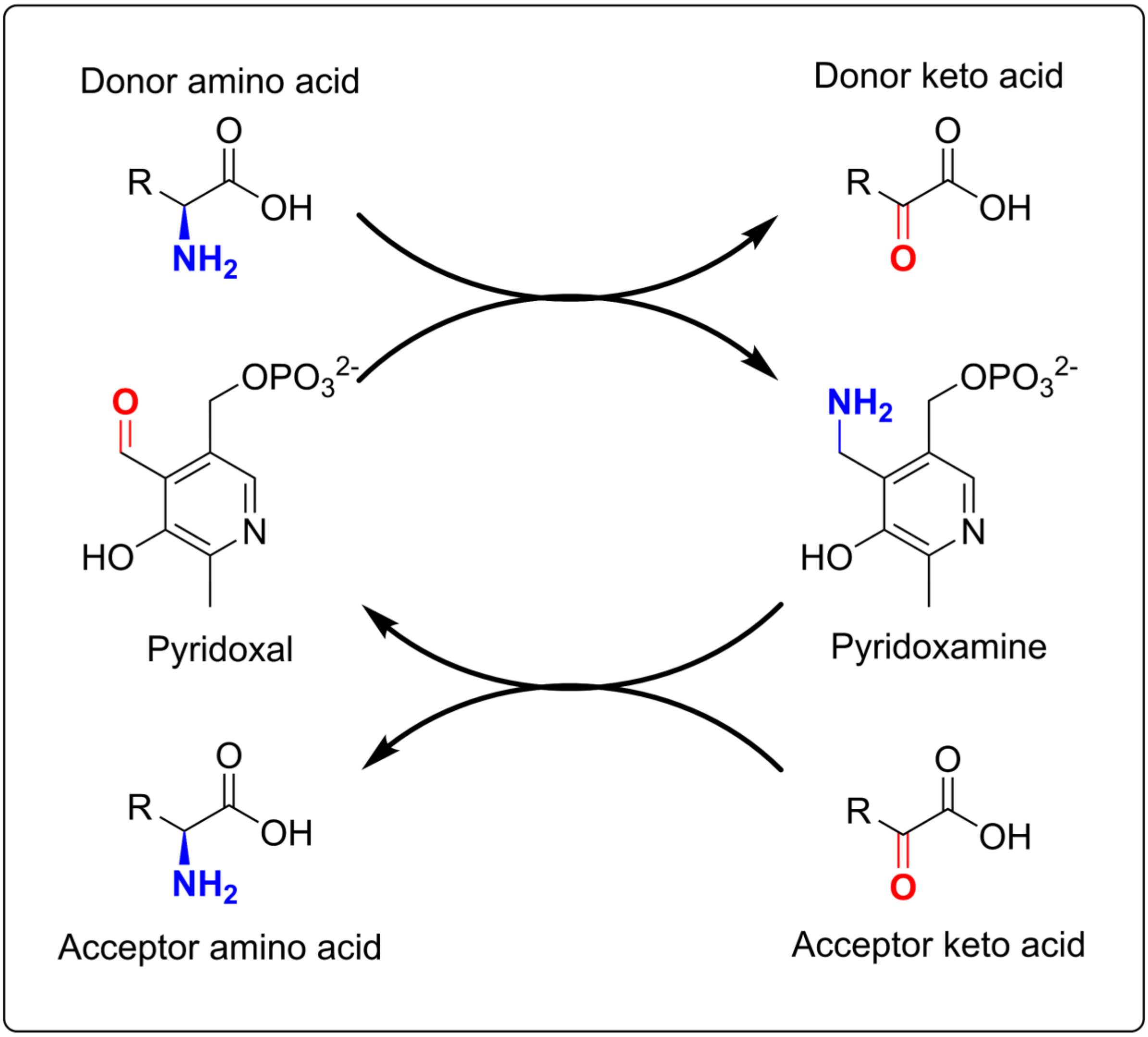

Figure S1: Transamination reaction cycle of amino acids with pyridoxal-5-phosphate that are observed in modern metabolism.
Amino groups have been coloured blue and α-keto groups in red to illustrate the reaction process taking place.

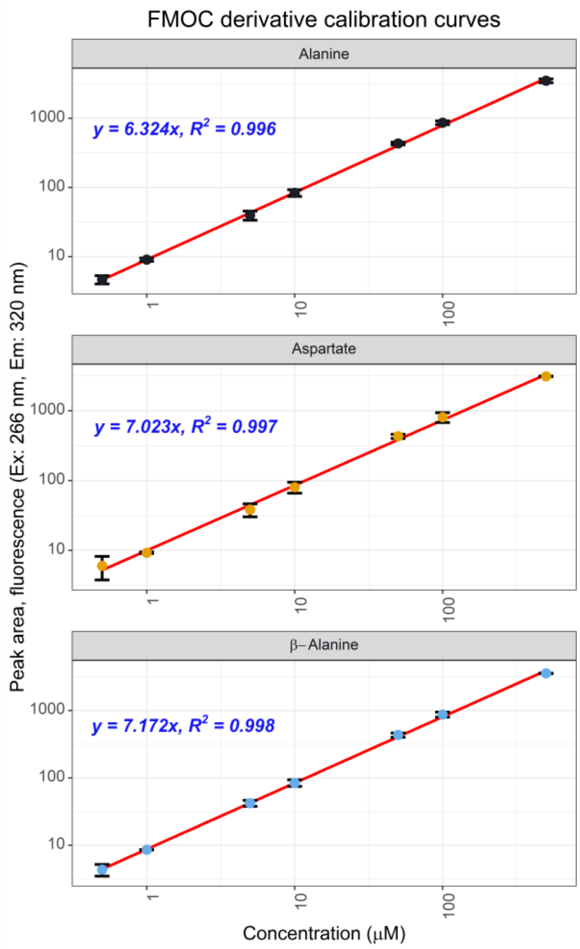

Figure S2. FMOC-amino acid calibration curves. $N = 3 \pm$ SD.

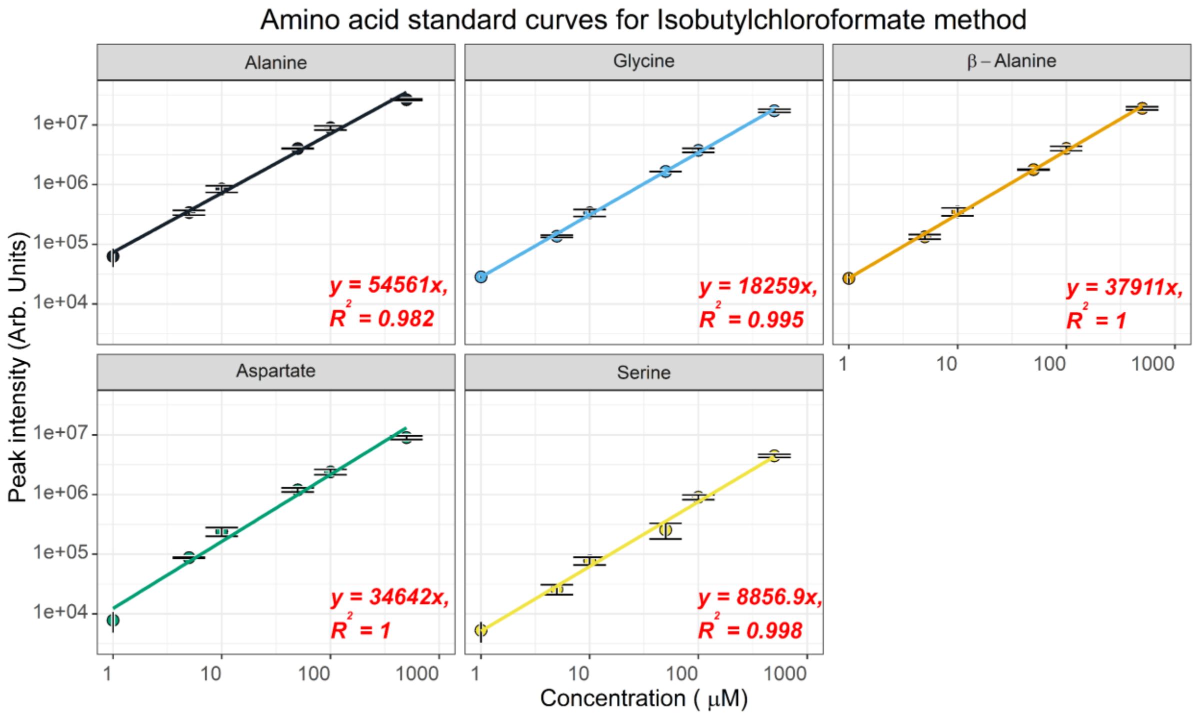

Figure S3. iBuCF-amino acid calibration curves for five amino acids. $N = 3 \pm$ SD.

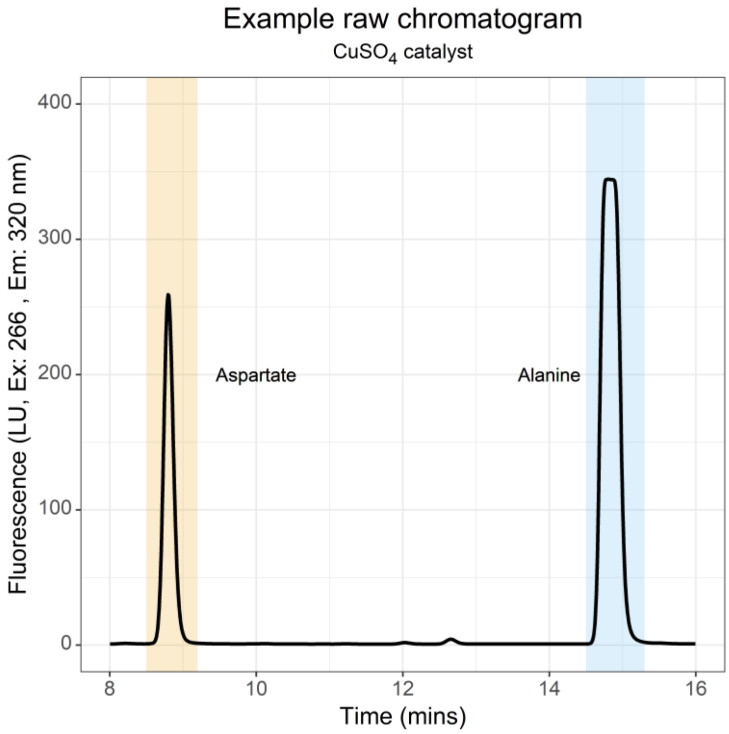

Figure S4. An example HPLC chromatogram from the cation screen (see section 3.1.1). The orange bar shows the aspartate-FMOC peak, the blue bar shows the alanine-FMOC peak. The squared peak shape of the alanine peak compared to the aspartate peak indicates likely detector saturation.

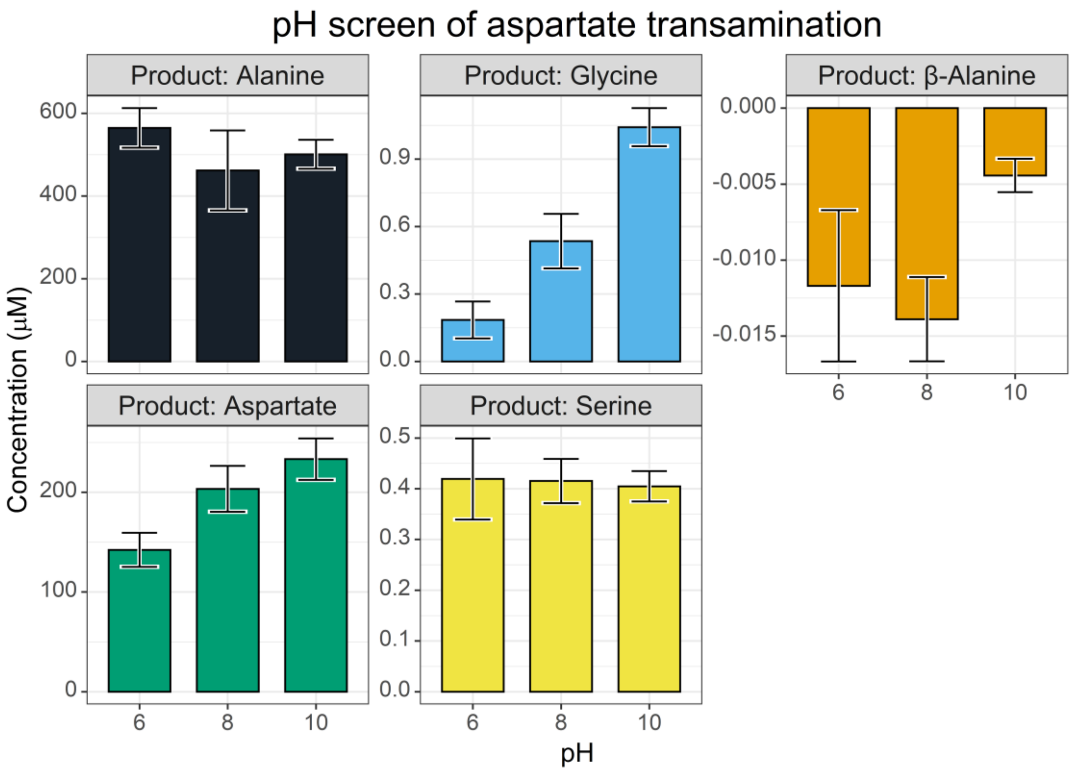

Figure S5. GC-MS verification for pH screen. Yields of selected amino acids were determined at the 1-hr time point for three different pH values via the iBuCF GC-MS method. Reactions were conducted with 10 mM pyridoxamine, 10 mM oxaloacetate in 50 mM ammonium bicarbonate with 1 mM $CuSO_4$ at 70 °C. Concentrations have been normalised relative to a water control. $N = 3 \pm$ SD.

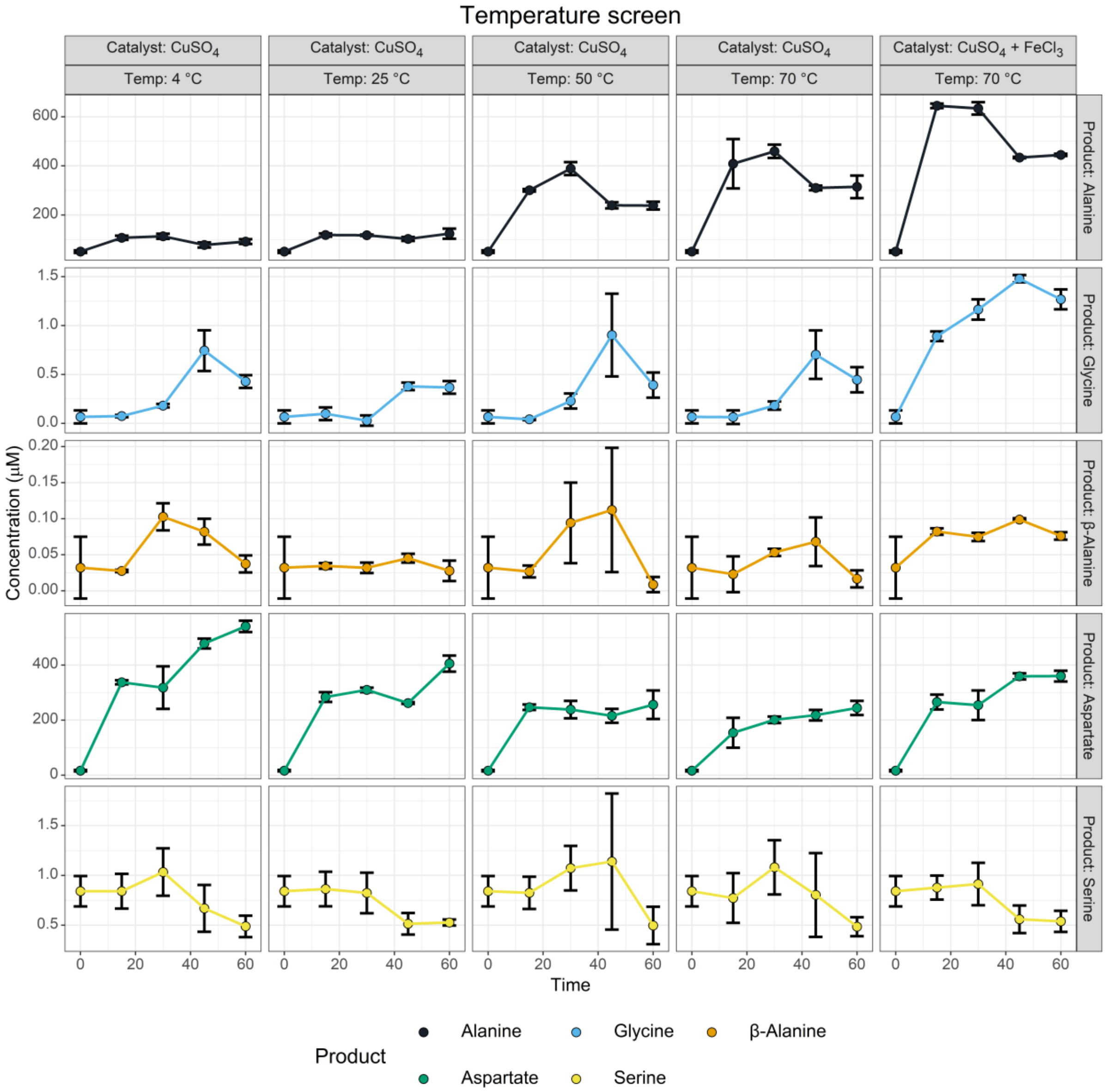

Figure S6. Temperature screen in expanded format. Yields of amino acids determined during a 1-hr reaction across five different temperature conditions via the iBuCF GC-MS method. Data are identical to those in Figure 6 but have been expanded to show the changes in amino acids with low yields. *N* = 3 ± SD.

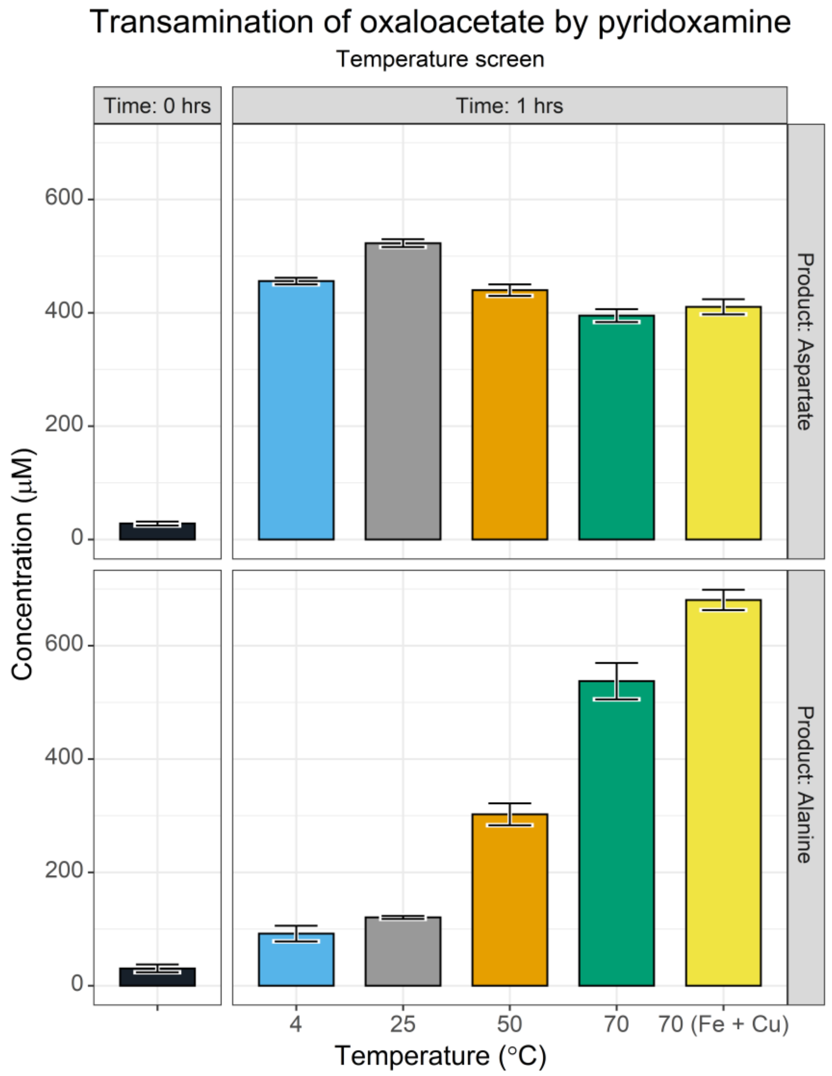

Figure S7. HPLC data for the temperature screen. Yields of aspartate and alanine determined during via the FMOC-HPLC method. Reactions were conducted with 10 mM pyridoxamine, 10 mM oxaloacetate in 50 mM ammonium bicarbonate (pH 7.8) with 1 mM $CuSO_4$ (with and without 1 mM $FeCl_3$) as required. T = 0 is common to all temperature screens. *N* = 3 ± SD.

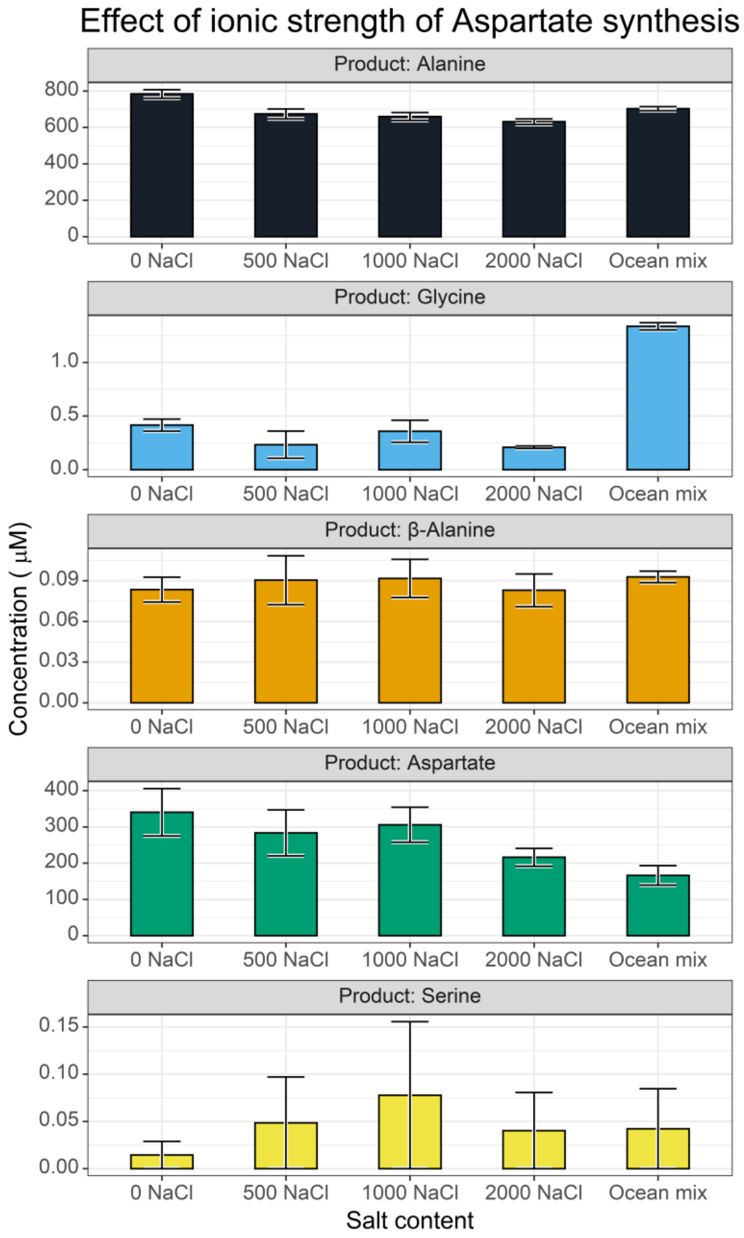

Figure S8. GC-MS verification of the ionic strength screen. Yields of selected amino acids after a 3-hour reaction as determined by the iBuCF GC-MS method. Reactions were conducted with 5 mM pyridoxamine, 5 mM oxaloacetate in 50 mM ammonium bicarbonate (pH 7.8) at 70 °C with 1 mM $CuSO_4$, and the appropriate salt mixture as required. $N = 3 \pm$ SD.

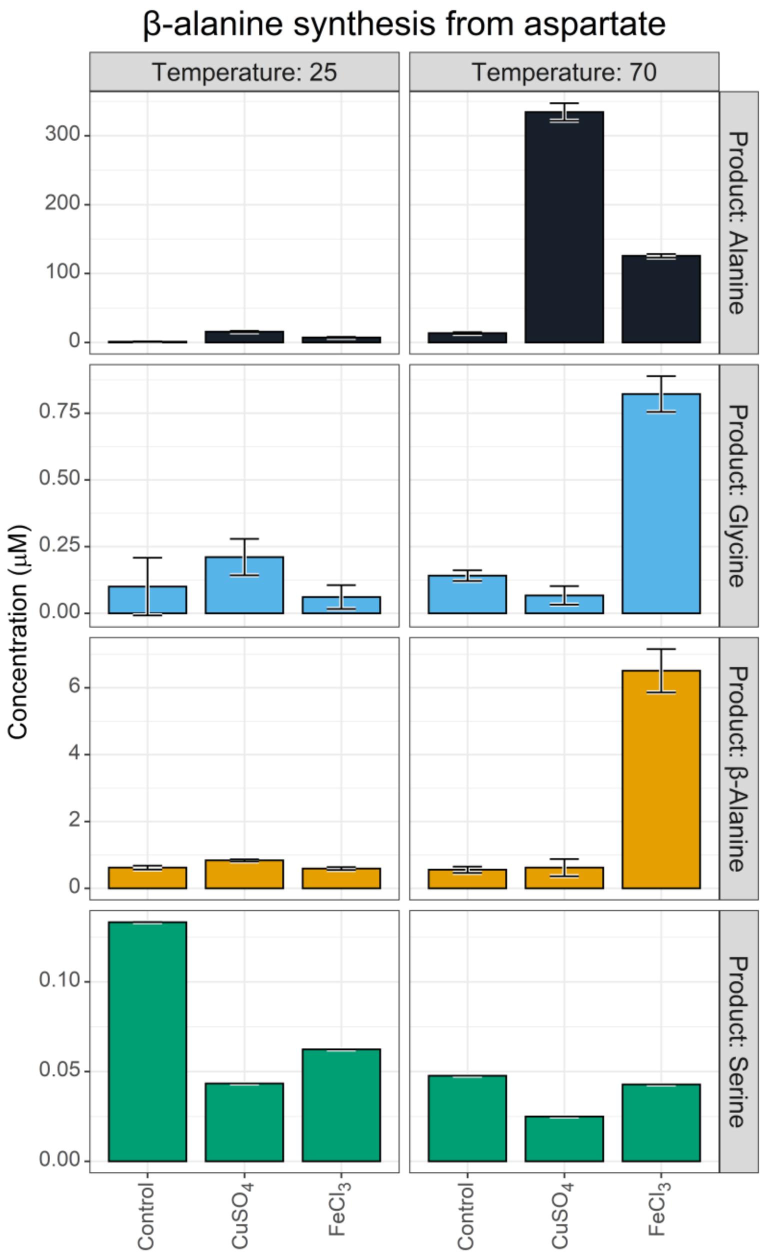

Figure S9. GC-MS verification of β-alanine synthesis from aspartate. Yields of selected amino acids after a 3-hour reaction as determined by the iBuCF GC-MS method. Reactions were conducted with 10 mM aspartate, 10 mM pyridoxal in 50 mM ammonium bicarbonate (pH 7.8) with 1 mM metal catalyst. *N* = 3 ± SD.
